# Leading in times of crisis: How perceived COVID‐19‐related work intensification links to daily e‐mail demands and leader outcomes

**DOI:** 10.1111/apps.12357

**Published:** 2021-12-02

**Authors:** Laura Venz, Katrin Boettcher

**Affiliations:** ^1^ Institute for Management and Organization Leuphana University Lüneburg Lüneburg Germany; ^2^ Department of Business and Economics Berlin School of Economics and Law Berlin Germany

**Keywords:** COVID‐19‐related acceleration, diary study, e‐mail overload appraisal, exhaustion, transformational leadership

## Abstract

The COVID‐19 crisis brought numerous challenges to work life. One of the most notable may be the acceleration of digital transformation, accompanied by an intensification of e‐mail usage and related demands such as high e‐mail workload. While research quickly started to examine the implications of these changes for employees, another important group of stakeholders has been overlooked: leaders. We focus on leaders during the COVID‐19 crisis and examine how COVID‐19‐related work intensification links to leaders' e‐mail overload appraisal and finally exhaustion and transformational leadership, a leader behaviour especially needed in times of crisis. In a 5‐day diary study in September 2020, 84 leaders responded to daily surveys on 343 days. Results of multilevel analysis showed that perceived COVID‐19‐related work intensification was positively linked to worktime spent dealing with e‐mail and appraised e‐mail overload. E‐mail overload appraisal was positively related to leaders' exhaustion, but unrelated to their transformational behaviour. Day‐specific time spent dealing with e‐mail, however, was negatively related to transformational leadership. E‐mail overload appraisal mediated the relationship between COVID‐19‐related work intensification and exhaustion. Turning the focus on leaders during the COVID‐19 crisis, our study has important implications for the design of work of leaders in times of crisis and beyond.

## INTRODUCTION

The world of work has changed in the wake of the COVID‐19 pandemic. For example, many started to work from home what brought changes in the way employees—and their leaders—collaborate and communicate (Rigotti et al., 2020). These changes became especially apparent in a shift towards intensified information communication technology (ICT) use, particularly e‐mail (DeFilippis et al., 2020). With this COVID‐19‐related acceleration of digital transformation (Kudyba, 2020), also ICT demands (Day et al., 2012) have intensified (e.g. Wang et al., 2021). This is likely to be true for everyone who is being part of the “digital workforce” (Colbert et al., 2016). Yet, compared to their followers, leaders might face even more (ICT) demands as they are generally held responsible to ensure good communication and performance (Barling & Cloutier, 2017; Dabbish & Kraut, 2006; Li et al., 2018; Sherf et al., 2019). Hence, leaders might be particularly challenged during crisis (Development Dimensions International, Inc., 2021; Haddon et al., 2015). This is where our study starts.

Already before the COVID‐19 crisis, leadership was communication‐intensive, having seen an increase in the use of e‐mail for work‐related communication (Rosen et al., 2019). Work e‐mail, in turn, has been coined “a source and symbol of stress” (Barley et al., 2011, p. 887) that likely limits leaders' chances both to be well and to lead well. Accordingly, the accelerated use of work e‐mail in the wake of the pandemic led to a “dramatic shift in leadership” with expected implications for leader well‐being and leader behaviour (Development Dimensions International, Inc., 2021, p. 3). We have subjected this assumption to an empirical test: In September 2020, six months after the pandemic hit, we conducted a diary study with a sample of leaders to, first, examine how leaders' perception of COVID‐19‐related work intensification relates to appraised e‐mail overload at work and, second, to investigate how e‐mail overload appraisal relates to leaders' exhaustion and transformational behaviour.

**FIGURE 1:**
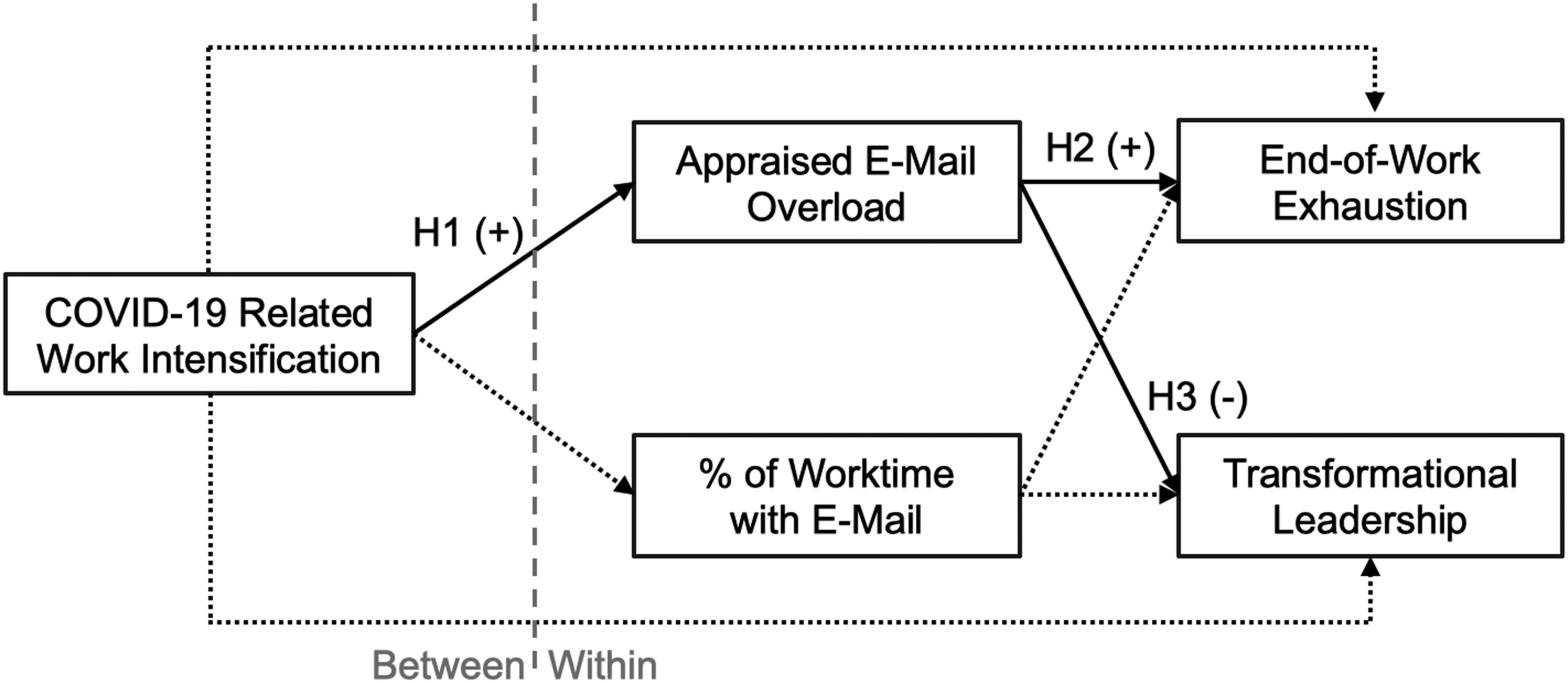
Path model of tested relationships. Note. All depicted within‐level paths were modelled at both the within‐person level and the between‐person level. Solid lines denote hypothesised paths. Dotted lines denote additionally modelled paths pertaining to the control variable. The dashed path from transformational leadership to end‐of‐work exhaustion was modelled in a supplementary analysis

In sum, we examine aspects that affect leaders in times of crisis, specifically how work intensification in the wake of the COVID‐19 pandemic affects leaders' perceived e‐mail demands, their well‐being, and their leadership behaviour. Integrating the model of contextual leadership (Oc, 2018) with the transactional model of stress (Lazarus & Folkman, 1984), we hypothesise that leaders' perception of COVID‐19‐related work intensification as a general stressor will be positively related to them perceiving e‐mail overload in their daily work. Furthermore, on days when leaders' e‐mail overload appraisal is higher, they will be more exhausted and behave less transformational (see Figure 1).

With our diary study during the COVID‐19 pandemic, examining leader well‐being and behaviour as two crucial outcome variables, we make three contributions. First, we examine if work intensification in the wake of the crisis is reflected in leaders' everyday work, specifically in their use of e‐mail and their e‐mail overload appraisal, respectively. Furthermore, we examine how day‐specific e‐mail overload appraisal in turn affects leaders' well‐being and behaviour. By putting our focus on leaders during the COVID‐19 crisis, we expand emerging research on work during the pandemic that, so far, rather focused on employees (e.g. Chong et al., 2020). Given the crucial role of leaders in times of crisis (e.g. Hu et al., 2020), this is an important advancement. At the same time, we meet calls for research that explicitly examines leaders' ICT use, considering the vital role that it likely plays in leader behaviour and thus follower outcomes (Wang et al., 2020). To obtain a reliable account of e‐mail overload, exhaustion, and transformational leadership and to do justice to the fact that day‐to‐day variation in e‐mail demands and leader behaviour has not been sufficiently considered in previous research (Rosen et al., 2019), we conducted a diary study.

Our second contribution is to consider leader well‐being in times of crisis, but also in general. In fact, the empirical literature is largely missing “any meaningful consideration of leaders' mental health” (Barling & Cloutier, 2017, p. 394), especially of leaders' *daily* well‐being (Sheridan & Ambrose, 2020). Our study contributes knowledge on the effects of the COVID‐19 crisis on leaders. In doing so, we advance knowledge on contextual factors that shape leaders' well‐being and on the toll day‐specific e‐mail demands exert on them (Rosen et al., 2019). Leaders' well‐being plays a critical role for their leadership behaviour and thus for their followers and organisations (Barling & Cloutier, 2017). Hence, additionally considering that many changes brought about by the pandemic, such as increased reliance on e‐mail, will last (Rigotti et al., 2021), it is crucial to understand the impact of e‐mail demands on leader well‐being (Wang et al., 2020). We contribute to this understanding.

Third, our study contributes to the surprisingly little knowledge about contextual predictors of transformational leader behaviour (Dóci & Hofmans, 2015). Given that transformational leadership is a particularly effective leader behaviour in relation to employee performance and well‐being (Nielsen & Taris, 2019), it is critical to understand how it emerges (Dóci et al., 2020). Our study contributes knowledge on this important issue. Again, our diary study approach is highly useful in this regard (see McCormick et al., 2020). Like any work behaviour, leader behaviour fluctuates from day to day (McClean et al., 2019). Such day‐to‐day variation in transformational leadership, in turn, has been shown to matter for positive employee outcomes (Kelemen et al., 2020). We add to the few existing diary studies that surveyed leaders in organisations and predicted their day‐specific transformational behaviour from contextual factors (Dóci et al., 2020; Nielsen & Cleal, 2011; Rosen et al., 2019).

Altogether, our study provides a constructive replication and extension (Köhler & Cortina, 2021) of previous findings on the effects of work intensification (e.g. Paškvan et al., 2016) and e‐mail overload (e.g. Brown et al., 2014). In the context of the COVID‐19 crisis, we conducted a diary study, focusing on leaders as an essential group of organisational stakeholders that has been largely overlooked in occupational stress research (Barling & Cloutier, 2017). As such, our study is also important from a practical point of view as it has implications for the design of work for leaders—in times of crisis and beyond.

## THEORETICAL BACKGROUND AND HYPOTHESES

The general work environment and the discrete work context are closely intertwined (Venz et al., 2020). As an example, general intensification of (technological) work might become apparent in higher time pressure, higher workload, and higher use of ICT for work‐related communication in everyday work (Chesley, 2014; Kubicek et al., 2015). These everyday (technological) work circumstances in turn influence behaviour and well‐being at work (Barber & Hu, 2020; Venz et al., 2020; Wang et al., 2020). The notion that the general context influences the discrete context of work, which in turn shapes daily processes at work, is also reflected in the contextual model of leadership (Oc, 2018). Precisely, the contextual model of leadership explicitly considers how context influences leadership, for example leader behaviour.

Crises, such as the COVID‐19 pandemic, form an important part of the general work context (Oc, 2018). Indeed, the measures to contain the spread of COVID‐19 brought numerous changes to people's general work environment. One of the most notable of these changes is maybe the accelerated digital transformation of work (Kudyba, 2020) that brought with it an increase in the use of e‐mail for work‐related communication (DeFilippis et al., 2020). With these changes, employees and leaders report that their workload (i.e. amount of work) has intensified (Wang et al., 2021). Notably, this acceleration of work in the wake of the COVID‐19 crisis reflects the general notion that socio‐economic and technological changes can be influential in driving work intensification, the need to work on an ever‐increasing number of tasks or at increasing speed (Kubicek et al., 2015; Paškvan & Kubicek, 2017).

Work intensification is an influential work stressor that creates exhaustion and other strain reactions (e.g. Franke, 2015; Korunka et al., 2015; Paškvan et al., 2016). This relationship can be explained along the lines of the transactional model of stress (Lazarus & Folkman, 1984), which aims to describe the mechanism through which stressors relate to strain outcomes (i.e. the physiological, psychological, and behavioural reactions to stressors; Jex & Beehr, 1991). Specifically, the transactional stress model specifies the stressor‐strain relationship as a dynamic process (Pindek et al., 2019) in which the personal evaluation (i.e. appraisal) of external stressors (e.g. work intensification, workload) plays a central role. Precisely, appraisal mediates the stressor‐strain relationship (Lazarus, 1993; Webster et al., 2011).

Different stressors can be appraised differently, as hindrance and/or as challenge (Searle & Auton, 2015). Hindrance appraisal involves the evaluation that the person's functioning, goals, and well‐being are threatened (Webster et al., 2011). For example, a person might appraise their workload (i.e. stressor) as being too large to be dealt with (i.e. overload appraisal as a form of hindrance appraisal). Hindrance stressors, such as work intensification (Korunka et al., 2015), and hindrance appraisal, respectively, are stressful experiences that positively relate to strain (e.g. Gonzalez‐Mulé et al., 2021; Pindek et al., 2019). Challenge stressors are stressful as well but besides their positive relationship with strain, they are also motivating what additionally connects them to positive outcomes such as performance (Crawford et al., 2010). For example, a person might appraise their workload as a challenge they want to master; this will lead them to invest effort into their work, what will consume their resources and result in strain (e.g. Pindek et al., 2019) but will also increase their performance (e.g. Mazzola & Disselhorst, 2019).

Work intensification has been shown to function as a hindrance stressor (and not a challenge stressor; Korunka et al., 2015) that individuals indeed appraise as hindrance rather than as challenge (Paškvan et al., 2016). Accordingly, in line with the transactional model of stress (Lazarus & Folkman, 1984), the effects of work intensification on employee strain were found to be mediated by hindrance appraisal (Paškvan et al., 2016). We draw on these findings and suggest that hindrance appraisal, precisely e‐mail overload appraisal, mediates the relationship of leaders' experienced COVID‐19‐related work intensification with exhaustion and transformational leadership.

Advancing previous research, we examine the effects of work intensification, precisely COVID‐19‐related work intensification, using a diary design. This enables us to examine if there are actual differences in the daily work context and the individual appraisal thereof, respectively, between leaders who differ in their perception of work intensification. That is, we link work intensification as an aspect of leaders' general work context to their discrete, everyday work context (see Oc, 2018). Specifically, considering that COVID‐19 has accelerated the digital transformation of work (Kudyba, 2020), we suggest that COVID‐19‐related work intensification will become apparent in leaders spending more of their worktime with e‐mail and hence in their appraisal of e‐mail overload, or the individually perceived inability to process the amount of e‐mail in the time available as a form of hindrance appraisal (Brown et al., 2014; Dabbish & Kraut, 2006). In other words, we suggest that work intensification as a feature of the general work environment manifests itself in high e‐mail demands (e.g. e‐mail workload as a discrete work event) that are likely to be evaluated negatively (Barber & Hu, 2020; Day et al., 2019; Reinke & Ohly, 2021), which will be reflected in e‐mail overload appraisal.

In sum, integrating the model of contextual leadership (Oc, 2018) with the transactional model of stress (Lazarus & Folkman, 1984), we propose that, first, leaders who perceive COVID‐19‐related work intensification (i.e. stressor) will perceive e‐mail overload in their everyday work (i.e. hindrance appraisal), and that, second, e‐mail overload appraisal has implications for leaders' day‐specific behaviour and well‐being (i.e. strain). This assumption is in line with research that found that work intensification affects critical employee outcomes, such as exhaustion, via individual hindrance appraisal on the one hand (Paškvan et al., 2016) and with findings that showed that high e‐mail volume relates to exhaustion via e‐mail overload appraisal on the other hand (Brown et al., 2014). Regarding the first path of this process, the stressor‐appraisal relationship, we hypothesise:

Hypothesis 1 There will be a positive relationship between leaders' perceived COVID‐19‐related work intensification and appraised e‐mail overload.

As noted, the transactional stress model (Lazarus & Folkman, 1984) suggests that strain reactions are the result of a dynamic process triggered by external stressors. In this process, individual stressor appraisal plays a central role such that it is not the stressor itself that fosters strain, but how the stressor is appraised (Webster et al., 2011). For example, it would not necessarily be the e‐mail workload (i.e. e‐mail volume or time spent dealing with e‐mail) that triggers exhaustion (Kushlev & Dunn, 2015), but rather the individual's perception of e‐mail overload (Brown et al., 2014; Dabbish & Kraut, 2006; Day et al., 2019; Reinke & Chamorro‐Premuzic, 2014).

Hindrance appraisal indicates that one's well‐being and important resources needed to deal with a stressor are threatened (Webster et al., 2011), leaving the person overwhelmed by the stressor. Hence, hindrance, or overload, appraisal is likely to affect especially exhaustion (e.g. Sessions et al., 2020), a state of being overextended and low on physical resources (e.g. Shirom & Melamed, 2006). In line with these notions, hindrance appraisal (e.g. Webster et al., 2011), and particularly perceived e‐mail overload (Reinke & Chamorro‐Premuzic, 2014), has been found to be positively related to exhaustion. To sum up, having to deal with too high stressors, for example generally intensified work or too many e‐mails on a given day, will result in hindrance appraisal such as feeling overwhelmed by the amount of e‐mail (i.e. overload appraisal). Hindrance appraisal in turn underlies the stressor‐strain relationship (Lazarus & Folkman, 1984; Searle & Auton, 2015), especially the stressor‐exhaustion relationship (e.g. Sessions et al., 2020; Webster et al., 2011).

Importantly, with its consideration of the stress process as something highly dynamic (Pindek et al., 2019), the transactional stress model (Lazarus & Folkman, 1984) suggests that the appraisal of stressors might not be stable but might vary within person, for example from day to day. Indeed, recent diary studies found that day‐specific hindrance appraisal mediates the within‐person relationship between day‐specific stressors (e.g. interruptions; Smith et al., 2020; time pressure; Kronenwett & Rigotti, 2020; ICT‐related workload after hours; Reinke & Ohly, 2021) and immediate strain reactions. Accordingly, backed up by meta‐analytical findings that show a positive within‐person relationship between daily stressors that have a‐priori been categorised as hindrance stressors and strain reactions (Pindek et al., 2019), we hypothesise that day‐specific e‐mail overload appraisal will be positively related to end‐of‐work exhaustion:

Hypothesis 2 There will be a positive within‐person relationship between e‐mail overload appraisal and end‐of‐work exhaustion.

We also suggest that e‐mail overload appraisal will be positively related to exhaustion at the between‐person level (e.g. Brown et al., 2014; Reinke & Chamorro‐Premuzic, 2014). Drawing on the transactional stress model, which poses appraisal as mediator in the stress process (Lazarus & Folkman, 1984; see also Searle & Auton, 2015), we consequently hypothesise that e‐mail overload appraisal will mediate the relationship between perceived COVID‐19‐related work intensification (i.e. stressor) and exhaustion (i.e. strain). This hypothesis is supported by findings that general work intensification relates to exhaustion via hindrance appraisal (Paškvan et al., 2016).

Hypothesis 3 There will be a positive between‐person indirect relationship between leaders' perceived COVID‐19‐related work intensification and end‐of‐work exhaustion via e‐mail overload appraisal.

We propose that e‐mail overload appraisal will also predict leader behaviour, specifically transformational leadership. Transformational leadership refers to leaders motivating their followers to move beyond expectations and self‐interest through inspiration, intellectual stimulation, and individualised support (Bass, 1999; Rafferty & Griffin, 2004). Transformational leaders communicate a vision and inspire their followers to share and follow that vision. They encourage their followers to be open to new ideas and ways of approaching problems and individually support each follower, considering their individual needs.

In non‐crisis times, transformational leadership is highly effective in ensuring follower performance, health, and well‐being (Arnold, 2017; Hoch et al., 2018; Nielsen & Taris, 2019). Besides, transformational leadership is highly effective—and yearned for by followers—also in times of crisis (Pillai, 2013). We conclude that transformational leadership is desirable leader behaviour to keep employees well and engaged during the COVID‐19 pandemic. However, we argue that really showing transformational behaviour might be particularly difficult for leaders in these times of crisis.

Specifically, we suggest that feeling overwhelmed by e‐mail (i.e. e‐mail overload appraisal) hinders transformational leadership. We base this assumption again on the transactional stress model (Lazarus & Folkman, 1984), according to which stressors trigger strain responses that can be physical or psychological but also behavioural in nature (Jex & Beehr, 1991; Searle & Auton, 2015). Indeed, hindrance stressors predict behavioural reactions, such as low performance, low organisational citizenship behaviour, and high counterproductive work behaviour, besides psychological strain responses (Mazzola & Disselhorst, 2019). In addition, leaders' general workload has been shown to be negatively related to their supportive leadership behaviour (Stein et al., 2020).

In line with the transactional stress model (Lazarus & Folkman, 1984), we again argue that hindrance appraisal explains these relationships. As noted previously, hindrance appraisal means that resources are threatened by facing a stressor. In response to resource threat, people tend to withdraw effort from resource‐demanding behaviours in order to protect the resources they still have (Chong et al., 2020; Giumetti et al., 2013). Transformational leadership is a resource‐demanding behaviour (Lin et al., 2019; Stein et al., 2020). Accordingly, we propose that hindrance appraisal will be negatively related to transformational leadership. This is in line with findings that hindrance appraisal is negatively related to performance for employees (LePine et al., 2016) as well as leaders (Sessions et al., 2020).

Based on the mentioned considerations and empirical results, we hypothesise that e‐mail overload appraisal will be negatively related to transformational leader behaviour. More precisely, considering the dynamic nature of appraisal processes (Reinke & Ohly, 2021) and transformational leadership (McClean et al., 2019), we propose a negative within‐person relationship. This hypothesis is supported by findings of other diary studies with leader samples that found day‐specific task complexity (Dóci & Hofmans, 2015) and, most important for our hypothesis, day‐specific e‐mail demands (Rosen et al., 2019) to be negatively related to same‐day transformational leadership. In a similar vein, day‐specific workload was found to be negatively related to leader justice behaviour towards followers—because leaders prioritised technical tasks over relational leadership tasks on days with high workload (Sherf et al., 2019). In line with the transactional stress model (Lazarus & Folkman, 1984), these results indicate that when leaders perceive to be overloaded with other tasks on a given day, for example with processing of e‐mail, they experience a lack of critical resources such as time and energy needed to engage in leadership tasks (Lin et al., 2019), such as transformational leadership. Accordingly, leaders' day‐specific overload appraisal should be negatively related to their engagement in transformational leadership on that day. This assumption is supported by findings that day‐specific hindrance appraisal negatively predicts day‐specific positive interpersonal work behaviour (Mitchell et al., 2019) and task performance (e.g. Ma et al., 2020). We hypothesise:

Hypothesis 4 There will be a negative within‐person relationship between e‐mail overload appraisal and transformational leadership.

Based on meta‐analytic findings that a high level of hindrance stressors is negatively related to performance and positive interpersonal behaviours (Mazzola & Disselhorst, 2019) as well as on findings of a negative relationship between hindrance appraisal and task performance on the between‐person level in a diary study (Ma et al., 2021), we suggest that e‐mail overload appraisal will be negatively related to transformational leadership also at the between‐person level. Consequently, again drawing on the mediation notion of the transactional stress model (Lazarus & Folkman, 1984), we hypothesise that e‐mail overload appraisal will mediate the relationship between COVID‐19‐related work intensification and transformational leadership.

Hypothesis 5 There will be a negative between‐person indirect relationship between leaders' perceived COVID‐19‐related work intensification and transformational leadership via appraised e‐mail overload.

## METHODS

Data collection for this study took place in September 2020 in Germany, about 6 months after the German government had implemented the first measures to contain the spread of COVID‐19, which included calls to work from home and to arrange work to reduce personal contacts as far as possible. Students recruited study participants as part of their empirical theses. Participants had to work in a leadership position with at least one direct report. To obtain reliable results on leaders' daily e‐mail demands and appraisal, leadership behaviour, and well‐being, we conducted a five‐day diary study (Gabriel et al., 2019). We limited the data collection to one daily measurement point (daily after work) over the course of one workweek to not tax participants too much in this already challenging time. Participants filled in a general survey upfront, about 1 week before taking the daily surveys. The daily surveys could be answered between 3 p.m. and midnight of each specific survey day. For every leader who participated in the full survey, we donated 1€ to a charitable health organisation.

From the originally registered 112 persons, 97 responded to the general survey and indicated that they are indeed in a leadership position. From those, in turn, 91 responded to a total of 350 workday surveys. To render within‐person variation in the day‐specific variables (i.e. e‐mail overload appraisal, exhaustion, and transformational leadership) possible, we included only data from leaders who responded to at least two daily surveys on days on which they had worked. Further, to ensure that leaders could have engaged in transformational leadership in principle, we included only days for which leaders reported that they had contact with their employees (e.g. in person or via e‐mail). This resulted in our final data set with responses from 84 leaders on 343 days (*M* = 4.08 days per leader).

Leaders in the final sample (41.7% female, 56.0% male, 2.4% no response) had a mean age of 42.95 years (*SD* = 11.94) and a mean job experience of 18.00 years (*SD* = 11.22). Their mean weekly worktime was 41.65 hours (*SD* = 7.16). They had leadership responsibility for an average of 22.01 employees (*SD* = 43.31). The majority had completed a university degree (78.6%). With regards to job titles and occupations, our sample was diverse, representing different hierarchical levels (e.g. CEOs, department heads, managing directors, team leaders, and self‐employed), working in different branches including finance, health and social care, retail, and manufacturing. All leaders reported to currently use e‐mail at work to some extent, ranging from about 10 to about 90% of their worktime (*M* = 39.6%, *SD* = 23.3).

Leaders included in the final sample (*N* = 84) did not significantly differ from those who had responded to the general survey but were not included in the final sample (*N* = 13) with regard to their weekly workhours (*M* = 41.64, *SD* = 7.16 vs. *M* = 44.92, *SD* = 9.79; *t*[95] = −1.46, *p* = .15). They, however, reported significantly lower perceived COVID‐19‐related work intensification (*M* = 2.79, *SD* = 1.11 vs. *M* = 3.60, *SD* = 1.05; *t*[95] = −2.45, *p* = .02). This must be kept in mind when interpreting our findings.

### Measures

The study was conducted online. We used short measures for the daily variables to reduce participant burden (Gabriel et al., 2019). A full list of items is presented at https://osf.io/jn2d5/. Participants answered all items on 5‐point scales ranging from 1 (*strongly disagree*/*not at all*) to 5 (*strongly agree/completely*). Table 1 gives an overview of the descriptive statistics of and correlations between the measures.

**TABLE 1:** Means, standard deviations, intraclass correlations, Cronbach's alphas, and correlations between study variables

		*M*	*SD*_w_	*SD*_b_	ICC	1	2	3	4	5
1	COVID‐19 work intensification	2.79		1.11		(.93)	.26*	.39**	.23*	.30*
2	E‐mail time^a^	3.82	1.35	2.38	.76		‐	.32*	−.00	.14
3	Appraised e‐mail overload	2.03	0.53	0.56	.53		.12	(.79)	.51**	.29^†^
4	Exhaustion	2.07	0.52	0.65	.62		.04	.26**	(.89)	−.06
5	Transformational leadership	3.13	0.69	0.53	.37		−.16*	−.07	−.04	(.77)

*Note*: *SD*w = within‐person level standard deviation. *SD*_b_ = between‐person level standard deviation. ICC = intraclass correlation coefficient (ICC1). Cronbach's alphas are displayed in brackets on the diagonal. Displayed correlations are standardised multilevel correlations calculated in Mplus 8.5. Correlations above the diagonal are person‐level correlations (*N* = 84 leaders). Correlations below the diagonal are day‐level correlations (*N* = 343 days).

^a^ 11‐point scale from 0 (0%) to 10 (100%).

^†^ *p* < .10.

* *p* < .05.

** *p* < .01.

#### COVID‐19‐related work intensification

We measured COVID‐19‐related work intensification in the general survey. We adapted the original German five‐item work intensification scale by Kubicek et al. (2015) to the pandemic context. We communicated to the participants that we were interested to learn something about possible changes in their work characteristics and circumstances that have occurred since the start of the COVID‐19 pandemic. Sample items are “Since the start of the COVID‐19 pandemic, one has more often to do two or three things at once (such as eating lunch, writing e‐mails, and talking on the phone)” and “Since the start of the COVID‐19 pandemic, it is increasingly rare to have enough time for work tasks.” Cronbach's alpha was .93. Notably, leaders in our sample largely differed in their perception of COVID‐19‐related work intensification (range 1.0 to 5.0, *M* = 2.79, *SD* = 1.11).

#### Appraised e‐mail overload

We measured day‐specific e‐mail overload appraisal with four items, three of which we adapted from the e‐mail overload scale of the ICT demands scale by Dabbish and Kraut (2006). We translated the items to German using a translation‐back translation procedure (Brislin, 1970) and adapted them to day‐specific measurement. A sample item is “Today, I found dealing with my e‐mail overwhelming.” We added one self‐developed item (“Today, the amount of e‐mail overwhelmed me”). Mean Cronbach's alpha was .79; ICC was .53.

#### End‐of‐work exhaustion

We measured end‐of‐work exhaustion with the five‐item physical fatigue subscale of the Shirom‐Melamed Burnout Measure (Shirom & Melamed, 2006), in a German version adapted to day‐specific assessment (Venz & Nesher Shoshan, 2021). Participants indicated how they “feel at the moment.” A sample item is “I feel tired.” Mean Cronbach's alpha was .89; ICC was .62.

#### Transformational leadership

We measured day‐specific transformational leadership with four items, each reflecting another dimension of transformational leadership: “Today, I voiced a clear understanding of where we, as a team, are going” (vision), “Today, I challenged my subordinates to think about old problems in new ways” (intellectual stimulation), “Today, I encouraged my subordinates to see changing environments as situations full of opportunities” (inspirational communication), and “Today, I behaved in a manner which was thoughtful of my subordinates' personal needs” (supportive leadership). We adapted the items from the transformational leadership scale proposed by Rafferty and Griffin (2004) and translated to German by Pinck and Sonnentag (2018). Mean Cronbach's alpha was .77; ICC was .37.

#### Control variable

To make sure that not sheer time spent dealing with e‐mail (e.g. Barley et al., 2011) but really overload appraisal explains exhaustion and transformational leader behaviour, we controlled for the daily percentage of worktime spent dealing with e‐mail. Participants indicated the percentage of their worktime that they had spent reading and writing e‐mail on that day on a 11‐point scale ranging from 0 (*0%*) to 10 (*100%*). ICC was .76.

#### Construct validity

To test discriminant validity of our measures, we conducted multilevel confirmatory factor analyses in Mplus 8.5. We specified a two‐level model with three within‐level factors (e‐mail overload appraisal, exhaustion, transformational leadership) and four between‐level factors (COVID‐19‐related work intensification, e‐mail overload appraisal, exhaustion, transformational leadership). This model,^1^ χ^2^ = 402.873, *df* = 191, *p* < .001, Scaling Correction Factor (SCF) = 0.9155, AIC = 11461.023, RMSEA = 0.057, CFI = 0.884, SRMR_within_ = 0.060, SRMR_between_ = 0.110, fit the data better than any alternative plausible model, for example, a model in which e‐mail overload appraisal and exhaustion items formed a common factor on both levels, χ^2^ = 694.228, *df* = 196, *p* < .001, SCF = 0.8617, AIC = 11680.400, RMSEA = 0.086, CFI = 0.727, SRMR_within_ = 0.097, SRMR_between_ = 0.156. We conclude that the measures capture distinct constructs.

### Analytical procedure

We conducted two‐level path modelling (see Preacher et al., 2010) in Mplus 8.5. To avoid variance conflation, we modelled all relationships that involve Level 1 (i.e. day‐specific) variables on both analytical levels. As a pure between‐person variable, COVID‐19‐related work intensification was included as a predictor at Level 2 only. Figure 1 gives an overview of all modelled relationships, including those involving the control variable.

## RESULTS

We tested all hypotheses simultaneously by specifying one overall two‐level path model with parallel paths at the within‐person level and at the between‐person level. Results are shown in Table 2. This model had an acceptable fit, χ^2^ = 5.861, *df* = 2, *p* = .05, CFI = 0.928, RMSEA = 0.075, SRMR_within_ = 0.038, SRMR_between_ = 0.055. At the day level (i.e. within person), this model explained 6% of the variance in exhaustion and 3% of the variance in transformational leadership. At the person level (i.e. between person), this model explained 31% of the variance in exhaustion, 12% of the variance in transformational leadership, 16% of the variance in appraised e‐mail overload, and 7% of the variance in time spent dealing with e‐mail. Because time spent dealing with e‐mail was measured using a different response scale, we report standardised estimates (est.; STDYX standardisation in Mplus) to enable easier interpretation.

**TABLE 2:** Results of the multilevel path model

	E‐mail time (%)			Appraised e‐mail overload			End‐of‐work exhaustion			Transformational leadership		
	Est.	*SE*	*z*	Est.	*SE*	*z*	Est.	*SE*	*z*	Est.	*SE*	*z*
*Between person*												
COVID‐19 work intensification	.26	.11	2.35*	.39	.11	3.68**	.06	.12	0.51	.21	.14	1.45
E‐mail time							−.17	.12	−1.34	.04	.14	0.27
Appraised e‐mail overload							.53	.13	3.95**	.20	.16	1.27
*Within person*												
E‐mail time							.01	.06	0.15	−.15	.07	−2.21*
Appraised e‐mail overload							.25	.07	3.60**	−.05	.08	−0.65
*R* ^2^ between	.07			.16			.31			.12		
*R* ^2^ within							.06			.03		

*Note*: *N*_leaders_ = 84. *N*_days_ = 343. est. = standardised multilevel estimate. *SE* = standard error. Displayed estimates result from one overall two‐level model test (i.e. all relationships were estimated in one model) in Mplus 8.5. *R* ^2^ were obtained from the standardised Mplus output.

* *p* < .05.

** *p* < .01.

Hypothesis 1 stated that there will be a positive between‐person relationship between perceived COVID‐19‐related work intensification and e‐mail overload appraisal. COVID‐19‐related work intensification was positively related to appraised e‐mail overload, est. = .39, *p* < .001, providing support for Hypothesis 1. Additionally, COVID‐19‐related work intensification was positively related to time spent dealing with e‐mail, est. = .26, *p* = .02.

Hypothesis 2 stated that there will be a positive within‐person relationship between e‐mail overload appraisal and end‐of‐work exhaustion. Day‐specific e‐mail overload appraisal was positively related to end‐of‐work exhaustion, est. = .25, *p* < .001. Thus, Hypothesis 2 was supported. Day‐specific time spent dealing with e‐mail was unrelated to end‐of‐work exhaustion, est. = .01, *p* = .88. At the between‐person level, results were comparable: E‐mail overload appraisal was positively related to exhaustion, est. = .53, *p* < .001, whereas time spent dealing with e‐mail was unrelated to exhaustion, est. = −.17, *p* = .18.

Hypothesis 3 suggested a positive between‐person indirect relationship between leaders' perceived COVID‐19‐related work intensification and exhaustion via e‐mail overload appraisal. We tested the indirect effect using the product of coefficient method (MacKinnon et al., 2007) based upon the unstandardised path estimates. We created the related confidence intervals using Monte Carlo simulation with 20,000 repetitions (Selig & Preacher, 2008). The between‐person indirect effect was significant, unstandardised indirect effect = .12, 95% CI [.03, .26]. Beyond the indirect effect, COVID‐19‐related work intensification was not directly related to exhaustion, est. = .06, *p* = .61.

Hypothesis 4 stated that there will be a negative within‐person relationship between e‐mail overload appraisal and transformational leadership. Day‐specific e‐mail overload appraisal was not significantly related to transformational leadership, est. = −.05, *p* = .52. Thus, Hypothesis 4 was not supported. Day‐specific time spent dealing with e‐mail, however, was negatively related to transformational leadership, est. = −.15, *p* = .03. At the between‐person level, neither appraised e‐mail overload, est. = .20, *p* = .20, nor time spent dealing with e‐mail, est. = .04, *p* = .78, was related to transformational leadership.

Hypothesis 5 suggested a negative between‐person indirect relationship between leaders' perceived COVID‐19‐related work intensification and transformational leadership via e‐mail overload appraisal. The between‐person indirect effect was not significant, unstandardised indirect effect = .04, 95% CI [−.02, .11]. COVID‐19‐related work intensification was not significantly directly related to transformational leadership either, est. = .21, *p* = .15.

### Additional analysis

Transformational leadership behaviour and end‐of‐work exhaustion might not occur independent of each other but might rather be causally related: Engaging in transformational leadership is resource demanding (Lin et al., 2019; Stein et al., 2020) and might thus generate exhaustion (Zwingmann et al., 2016). In an additional analysis, we therefore examined if transformational leadership predicts end‐of‐work exhaustion. We adapted our path model by adding a direct path from transformational leadership to exhaustion on both levels (see Figure 1). Transformational leadership was unrelated to end‐of‐work exhaustion within person, est. = −.02, *p* = .70, but was negatively related to exhaustion between persons, est. = −.24, *p* = .02.

## DISCUSSION

The COVID‐19 pandemic served as an accelerator of digital transformation (Kudyba, 2020), accompanied by an intensification of e‐mail usage. We examined which role COVID‐19‐related work intensification plays for leaders' daily work in times of the COVID‐19 crisis. Specifically, based on the model of contextual leadership (Oc, 2018) and the transactional model of stress (Lazarus & Folkman, 1984), we tested how COVID‐19‐related work intensification links to leaders' daily appraisal of e‐mail overload and how e‐mail overload appraisal, in turn, links to leaders' exhaustion and transformational leadership.

The nature and severity of the changes that the COVID‐19 pandemic has brought about differ between regions, organisations, and industries (DeFilippis et al., 2020). Accordingly, it is not surprising that leaders in our sample largely differed in their perceived COVID‐19‐related work intensification. The extent to which leaders perceived that COVID‐19 had intensified their work, though, was indeed positively related to their e‐mail usage and appraised e‐mail overload measured daily over the course of one workweek. At the day (i.e. within‐person) level, in turn, on days when e‐mail overload appraisal was high, leaders were more exhausted at the end of the day. Likewise, at the between‐person level, leaders who reported higher e‐mail overload appraisal indicated to be more exhausted than leaders who reported lower e‐mail overload appraisal. Overload appraisal mediated the relationship between COVID‐19‐related work intensification and exhaustion. E‐mail overload appraisal was unrelated to leaders' transformational behaviour at both analytical levels. Yet, on days when leaders spent more time dealing with e‐mail, they reported lower transformational leadership behaviour. Transformational leadership and end‐of‐work exhaustion were unrelated to each other within person (i.e. on the day level) but were negatively related between persons.

### Theoretical and empirical implications

Our study has several implications for theory and research. First, our study contributes to research on work intensification. Our results, which we obtained in the context of the COVID‐19 crisis, show that global crises can relate to persons' perception of work intensification. In our study, perceived COVID‐19‐related work intensification was reflected in leaders' discrete work context in terms of both higher e‐mail usage and higher e‐mail overload appraisal. This finding necessitates a need for more scholarly attention to the role that the general work context, including societal and technological factors, plays in everyday work (see Oc, 2018).

At the same time, our study points to the usefulness to consider individual perceptions of contextual factors beyond more objective indicators. For example, although all leaders in our sample were facing the COVID‐19 crisis, their individual perception of work intensification in the wake of the crisis differed considerably. Kubicek et al. (2015) reported similar results, namely that people largely differ in their perception of general work intensification. Further, the authors reported a positive relationship between the frequency of ICT use and perceived work intensification. We expanded this finding in the context of the COVID‐19 pandemic looking at how perceived work intensification becomes apparent in everyday work. Leaders who perceived work intensification spent more time dealing with e‐mail at work and appraised higher e‐mail overload. Hence, the perceived intensification of work is reflected in higher demands and more negative evaluation (i.e. hindrance appraisal) of demands in everyday work.

Our study further suggests that work intensification negatively affects leaders in their everyday work: e‐mail overload appraisal mediated the effects of work intensification on leaders' end‐of‐work exhaustion. This finding supports the notion that the stress process, especially individual appraisal, is dynamic and that this dynamic matters in explaining well‐being (Lazarus & Folkman, 1984; Pindek et al., 2019; Reinke & Ohly, 2021). In sum, our study supports that work intensification is a critical work stressor (e.g. Franke, 2015; Korunka et al., 2015; Paškvan et al., 2016) that, as a factor of the general work context, shapes leaders' discrete, day‐to‐day work context (see Oc, 2018) with implications for their well‐being and functioning at work.

In this regard, another contribution of our study is that it takes leaders' well‐being (Barling & Cloutier, 2017) and transformational leadership behaviour (Rosen et al., 2019) in relation to their e‐mail usage into consideration (Wang et al., 2020). With our focus on within‐person processes, our study advances the understanding of factors that shape leaders' dynamic well‐being and behaviour, especially on the toll contextual demands exert on leaders (Dóci et al., 2020; Nielsen & Cleal, 2011; Rosen et al., 2019). Notably, we expand the criterion space for research on day‐specific work context predictors of leader outcomes by leader exhaustion and show that different critical day‐specific leader outcomes have different predictors.

Regarding leader exhaustion, day‐specific time spent dealing with e‐mail was unrelated to, but appraised e‐mail overload was positively related to leaders' end‐of‐work exhaustion. This finding supports one of the core notions of the transactional model of stress (Lazarus & Folkman, 1984), namely that it is the individual appraisal and not the external demand that matters for the emergence of psychological strain. Kushlev and Dunn (2015) summarised that neither e‐mail volume nor time spent dealing with e‐mail seems to affect well‐being directly. Our study adds to recent studies that considered dynamic appraisal processes in relation to work‐related stressors and well‐being (e.g. Reinke & Ohly, 2021; Smith et al., 2020) and work behaviour (e.g. Ma et al., 2021; Mitchell et al., 2019) and replicates the effects of appraised e‐mail overload on exhaustion (e.g. Brown et al., 2014) at the within‐person level. We encourage other researchers to build on these findings and pay more attention to dynamic appraisal processes as well as to leaders' daily well‐being and to how it emerges.

Regarding leaders' daily transformational behaviour, our findings were opposite to those for exhaustion: day‐specific e‐mail overload appraisal was unrelated, but time spent dealing with e‐mail was negatively related to transformational leadership. This contradicts the theoretical assumption that appraisal but not the stressor itself fosters strain responses (Webster et al., 2011). Maybe, for day‐specific leader *behaviour*, it is more the actual availability of resources that underlies the process. Indeed, others have argued that transformational leadership consumes and thus requires resources (Dóci et al., 2020; Stein et al., 2020). However, it is rather unclear which exact resources leaders need to have available to be able to engage in dynamic transformational leadership. Although we suppose that both personal resources, such as positive self‐evaluations (Dóci et al., 2020) and personal energy (e.g. Barnes et al., 2016), and contextual resources, especially time (Stein et al., 2020), will be important preconditions for day‐specific transformational behaviour, we speculate that time might play an even a larger role than personal energy in this regard. We surmise that even if a leader has the energy resources available to be potentially invested in transformational leadership, if they lack the time to be transformational, they might invest these resources into other behaviours (see Stein et al., 2020). When facing high e‐mail volume on a given day, leaders must make choices on how they divide their time between processing e‐mail and other activities, such as leadership behaviour (Rosen et al., 2019). In this case, leaders might rather invest their available resources in processing their e‐mail, because it might be more salient and will thus be prioritised over leadership behaviours (Sherf et al., 2019). Future research might try to shed light on the resource processes that underlie the within‐person relationship between leaders' (quantitative) e‐mail demands and their leadership behaviour.

Finally, our study also has research implications for the measurement of e‐mail demands, or more broadly ICT usage at work (see Day et al., 2019). We encourage scholars to distinguish discrete ICT events (Barber & Hu, 2020), for example sheer e‐mail volume or processing time, from their appraisal. Notably, in our study, time spent dealing with e‐mail was unrelated to e‐mail overload appraisal at the day level, what mirrors previous between‐person results that did not find a relationship between e‐mail quantity and overload appraisal either (Reinke & Chamorro‐Premuzic, 2014). Therefore, both aspects need to be explicitly considered when measuring e‐mail demands. Given the paradoxical nature of ICT usage with parallel negative and positive effects (Day et al., 2019), future studies should also include challenge appraisal (see Searle & Auton, 2015; Webster et al., 2011) of e‐mail demands.

In sum, our study extends theory and research by adopting a within‐person view (Gabriel et al., 2019; McCormick et al., 2020) on how e‐mail demands affect leaders' daily well‐being and behaviour (Barber & Hu, 2020; Rosen et al., 2019). With our study, conducted in times of the COVID‐19 crisis, we were able to constructively replicate and extend (Köhler & Cortina, 2021) previous findings on the detrimental effects of work intensification on exhaustion (e.g. Korunka et al., 2015; Paškvan et al., 2016), considering appraisal and exhaustion in everyday work as mechanism and outcome, respectively. In a similar vein, we moved findings on the critical role of e‐mail overload appraisal (e.g. Reinke & Chamorro‐Premuzic, 2014) to the within‐person level, considering that the stress and appraisal process is inherently dynamic (Pindek et al., 2019; Reinke & Ohly, 2021). Importantly, because higher‐level factors such as the general work environment or individual differences are implicitly controlled for in the within‐person level analyses (Gabriel et al., 2019), our study has implications for theory and research beyond the context of crises. We support the call of Rosen et al. (2019, p. 28) to pay more attention to e‐mail demands and transformational leadership behaviour as dynamic phenomena but would like to extend this call to also point out the need to research leader well‐being at the day level.

### Limitations and future directions

Without wanting to downplay its methodological strengths, namely the diary design surveying organisational leaders in the wake of the COVID‐19 pandemic, our studies' limitations must also be mentioned and considered in interpreting the results. First, dropout analyses showed a significant difference in COVID‐19‐related work intensification perceptions between those leaders who filled in at least two of the daily surveys (and were thus included in the final sample) and those who did not. Hence, our findings might suffer from range restriction and related biased estimates (see Sackett & Yang, 2000). In other words, the real effect of COVID‐19 on leaders' e‐mail usage and overload appraisal and ultimately exhaustion and transformational leadership might be even larger than what we found. The fact that those who appear to have been affected most by the pandemic dropped out of our sample might not only have implications for our study but might be an important issue to be considered in all organisational research during crises: There is a threat to underestimate the negative effects that crises have on working people if those most affected are not part of the samples studied.

Second, our within‐person findings are based on cross‐sectional (albeit day‐specific) measurements and all data is self‐reported. Thus, we cannot preclude that our results suffer from same‐source bias (Podsakoff et al., 2012) and we cannot make causal claims. However, we measured our main predictor, COVID‐19‐related work intensification, before the other constructs, ensuring temporal separation in this regard. Further, exhaustion—the outcome variable for which we found our hypotheses supported—was measured in relation to the moment of responding to the daily survey but e‐mail time and appraised e‐mail overload related to the whole day at work. Accordingly, we enabled psychological temporal separation here. Finally, modelling the within‐person level relationships simultaneously at the between‐person level removed potential between‐person confounds regarding these relationships (see Gabriel et al., 2019).

For future studies, researchers who want to replicate our results might use several daily measurement points, such as in the morning, at noon, and after work, to be able to separate measurements of different variables to get closer to causal claims. Such future studies might additionally use daily follower ratings of transformational leadership, although the leaders themselves might in fact be the most‐reliable source of their day‐specific behaviour across all followers (cf. McClean et al., 2019). Finally, about measuring e‐mail demands, scholars might consider using objective measures, such as computer‐recorded time spent dealing with e‐mail or number and length of e‐mails received and written. However, when conducting research in real organisational settings, scholars need to consider that doing so could lead to participants' perception of being controlled as well as raise data protection and data security concerns (e.g. in terms of the content of e‐mails). In any case, participants' subjective perception and appraisal of their work context needs to be considered as well, as our findings indicate.

Besides replicating and extending our findings, future studies could address some of the new research questions our study raises. For example, future studies might explore in which industries and for which leaders (e.g. specific leadership role or hierarchical level) work intensification and high e‐mail demands are especially prevalent or problematic. Furthermore, it might be interesting to examine how leader exhaustion and transformational leadership are related to each other at the day level. In our study, transformational leadership and end‐of‐work exhaustion were not related within person. This result differs from longitudinal between‐person findings that show transformational leadership to positively predict leaders' exhaustion over time (Lin et al., 2019; Zwingmann et al., 2016). Other studies yet found the reversed relationship with exhaustion negatively predicting transformational leadership over time (e.g. Walsh & Arnold, 2018). Combining these findings with our results, it might be that leaders use transformational leadership only on days when their energy resources are sufficient to both pursue their salient tasks (e.g. process their e‐mail) and engage in transformational leadership. Thus, available energy might be a necessary precondition for leaders to engage in day‐specific transformational behaviours (see Sheridan & Ambrose, 2020). It might even be that when specific demands are pressing (e.g. high e‐mail volume), leaders might not simply refrain from transformational leadership but in fact cut back transformational behaviours (see Trougakos et al., 2015, for a similar reasoning) to save energy resources from being overused (see Venz & Nesher Shoshan, 2021, for an example of such a mechanism of day‐specific coping). Considering these ideas, we deem it interesting for future studies to shed light on the role that state exhaustion plays in predicting dynamic leader behaviour, how exhaustion and transformational leadership are related reciprocally within and between days, and what effects day‐specific e‐mail demands and their appraisal have in this conjunction. Experience sampling studies with multiple daily measurement points seem well suited to examine these questions. Such studies might additionally broaden the scope of examined constructs by testing the role of other personal resources besides energy (e.g. core self‐evaluations; Dóci et al., 2020) as well as contextual resources (e.g. time; see also Oc, 2018) in these dynamic relationships.

### Practical implications

The Global Leadership Forecast 2021 (Development Dimensions International, Inc., 2021), which was published after our data collection, found that 60% of the leaders surveyed during the COVID‐19 pandemic felt exhausted at the end of every workday. Further, 80% of the leaders stated that they lack confidence in leading virtually and 71% reported that they do not feel prepared to efficiently work in a digital environment. These numbers show how important it is to take leaders' ICT demands, well‐being, and behaviour during the pandemic into consideration. In this regard, our results provide several insights on managing leaders' well‐being and leadership behaviour in the COVID‐19 crisis—and beyond.

To start with, we caution organisations to not ignore the potential negative implications that the COVID‐19‐related acceleration of digital transformation (Kudyba, 2020) might have. Our results give first indications on that as e‐mail communication increases leaders might reduce their transformational leadership behaviour. Considering that transformational leadership is highly effective to ensure employee well‐being and performance both in times of crisis (Pillai, 2013) and in times of non‐crisis (Arnold, 2017; Nielsen & Taris, 2019), organisations should make sure to not let the digital transformation—being it COVID‐19‐related or in general—to unhindered intensify leaders' work, especially their daily e‐mail demands. As our results indicate, this is not only critical to ensure positive leadership, but also to preserve leader well‐being, a good worth protecting for itself (Barling & Cloutier, 2017).

Given that reliance on e‐mail for work communication will likely stay a central element of leaders' work, though, organisations and leaders should also adopt practices that make work e‐mail manageable (see Barber & Hu, 2020; Day et al., 2019). To this end, both work design (see Wang et al., 2020) and leaders' individual resources and self‐management should be addressed. Since we found detrimental effects of sheer time spent dealing with e‐mail and of appraised e‐mail overload—for the first on transformational leadership and for the latter on exhaustion—both e‐mail quantity and appraisal processes must be considered to ensure both leader well‐being and positive leader behaviour. To reduce e‐mail quantity or the time spent dealing with e‐mail, organisational norms need to be established that allow sufficient time to respond to e‐mails. Realistic expectations for e‐mail responding times should be set and made explicit. Overall, e‐mail should not have higher priority than good leadership, at least not generally. Regarding the individual appraisal of e‐mail overload, efficient self‐regulation should be fostered. For example, leaders are well advised to check their e‐mail less frequently and only at specific, previously set and communicated times (Kushlev & Dunn, 2015; Reinke & Chamorro‐Premuzic, 2014).

## CONFLICT OF INTEREST

The authors declare that they have no conflict of interest.

## ETHICS STATEMENT

The present research is in line with the ethical guidelines of the German Psychological Society.

## Data Availability

The data that support the findings of this study are available on request from the corresponding author. The data are not publicly available due to privacy or ethical restrictions.

## ENDNOTE

^1^
The non‐optimal fit of this model is likely caused by the measure of transformational leadership on the person level, with the item “Today, I behaved in a manner which was thoughtful of my subordinates' personal needs” showing a factor loading of .46 (*p* = .02). Accordingly, results including transformational leadership at the person level need to be interpreted with caution. A sole within‐level CFA (3 factors: e‐mail overload, exhaustion, transformational leadership) fit the data well, χ^2^ = 159.151, *df* = 62, *p* < .001, RMSEA = 0.069, CFI = 0.925, SRMR_within_ = 0.054.
